# An incidental finding of retroperitoneal paraganglioma during the coronavirus disease 2019 pandemic: a case report

**DOI:** 10.1186/s13256-022-03513-5

**Published:** 2022-07-21

**Authors:** K. Ben Hamida, M. Slimane, A. Mlouka, N. Boujelbene, S. Essghaier, K. Rahal

**Affiliations:** 1 Department of Surgical Oncology, Salah Azaiez Institute, Boulevard du 9 Avril 1938, 1006 Tunis, Tunisia; 2 Department of Radiology, Salah Azaiez Institute, Boulevard du 9 Avril 1938, 1006 Tunis, Tunisia; 3 Department of Pathology, Salah Azaiez Institute, Boulevard du 9 Avril 1938, 1006 Tunis, Tunisia; 4 Faculty of Medicine, University of Tunis El Manar, Tunis, Tunisia

**Keywords:** COVID-19, Incidental finding, Paraganglioma, Retroperitoneum

## Abstract

**Background:**

Extra-adrenal paraganglioma of the retroperitoneum is a very rare neoplasm arising from cells of the primitive neural crest. Although paragangliomas are considered benign and are often found incidentally, they have the potential to metastasize.

**Case presentation:**

We report the case of a 68-year-old Caucasian woman with an incidental diagnosis of retroperitoneal paraganglioma that was discovered on chest computed tomography performed for high suspicion of coronavirus disease 2019 pneumonia. The patient showed no metastasis and was successfully treated by complete surgical removal of the tumor.

**Conclusion:**

As the diagnosis of paragangliomas is often delayed because of absent clinical symptoms, they represent a significant diagnostic challenge. Although surgery may exacerbate coronavirus disease 2019 infection, surgical resection of this tumor is prioritized, given its malignancy potential, and it must be performed as soon as no infection is detected.

## Background

Paragangliomas are tumors that arise from extra-adrenal medullary neural crest derivatives. Usually located in the head and neck, they can be found in various body sites, including the chest cavity, abdomen, pelvis, and bladder.

They can occur at any age, most commonly in young adults. Asymptomatic paragangliomas are less common than symptomatic ones.

Many cases of clinically asymptomatic paragangliomas are not found until they have grown comparatively large or were discovered incidentally.

To the best of our knowledge, no incidental finding of retroperitoneal paraganglioma during the coronavirus disease 2019 (COVID-19) workup has been reported in the literature.

We aim through this study to report our experience.

## Case presentation

A 68-year-old female Caucasian patient was transferred to our department in April 2021 after discovering a retroperitoneal mass. The patient’s history was unremarkable, except for mild arterial hypertension well treated with calcium channel blockers and diabetes. There were no hypertensive crises in her anamnesis.

**Fig. 1:**
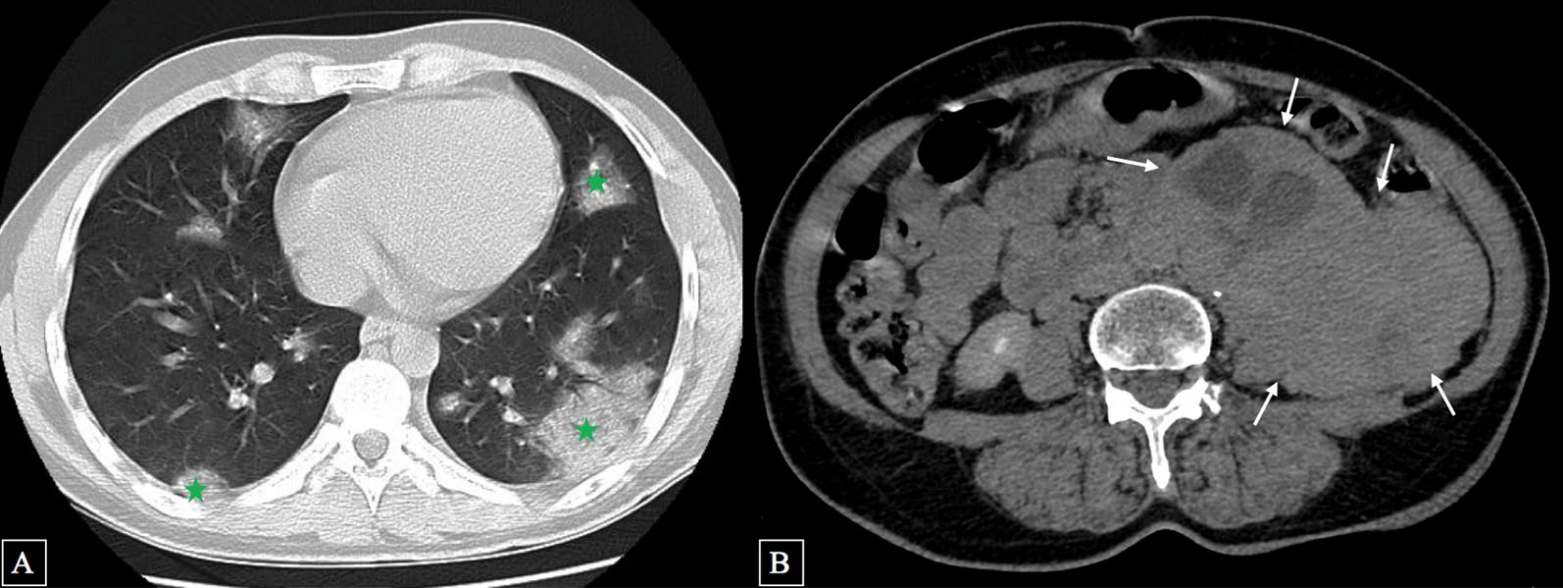
Computed tomography (CT) scan in pulmonary (**A**) and mediastinal (**B**) windows revealing ground-glass opacities related to COVID-19 infection (  ) and an incidental retroperitoneal paraganglioma (white arrow)

One month ago, she complained of dyspnea, fever, and chest pain. Given the presentation during the coronavirus pandemic, a computed tomography pulmonary angiography (CTPA) was performed (Fig. 1) in line with the diagnostic pathway for COVID-19, which incidentally showed a retroperitoneal necrotizing mass.

**Fig. 2.**
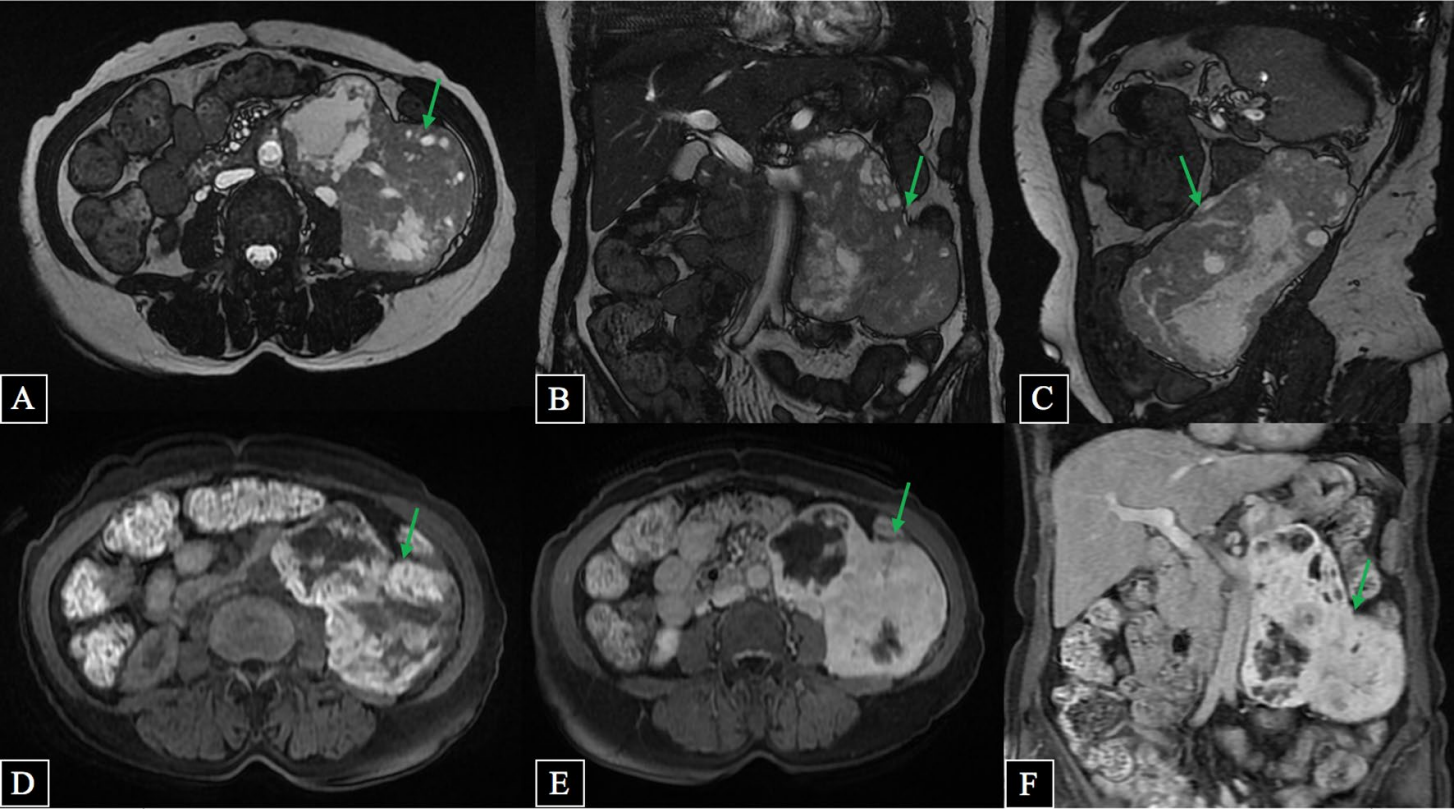
MRI in axial, coronal, and sagittal T2w (**A**–**C**), axial T1w (**D**), and axial and coronal T1 Gd (**E**, **F**) showing large retroperitoneal paraganglioma (  )

Subsequent magnetic resonance imaging (MRI) was performed (Fig. 2) and revealed an inhomogeneous, encapsulated, partially and vividly contrast-enhancing tumor, with a diameter of 16 × 12 cm. It was located in the left retroperitoneal with a close connection to the abdominal aorta, iliopsoas muscle, and upper pole of the left kidney. Thick and tortuous arteries and veins were observed inside and at the periphery of the tumor. There were no metastases or abdominal lymphadenopathy.

Retroperitoneal paraganglioma was then suspected. Special blood analysis for catecholamines was performed with the following results: noradrenaline (blood plasma) 3.57 nmol/l (norm value < 1.05 nmol/l), adrenaline (blood plasma) 0.48 pg/ml (norm value < 0.36 ng/l).

Urinary specimens were collected for assay 24-hour urine metabolites of catecholamines. The results were as follows: metanephrine: 326 µg/24 hours (norm value < 28 µg/24 hours), normetanephrine 1756 nmol/24 hours (norm value < 143 nmol/24 hours).

Immunohistochemical examination confirms neuroendocrine differentiation with chromogranin A 14,800 ng/ml (norm value < 104 ng/ml).

Our final diagnosis was a retroperitoneal paraganglioma with no evidence of metastatic spread.

After a multidisciplinary meeting, we decided to perform an open surgery preceded by a preoperative antihypertensive medication for safe resection.

**Fig. 3:**
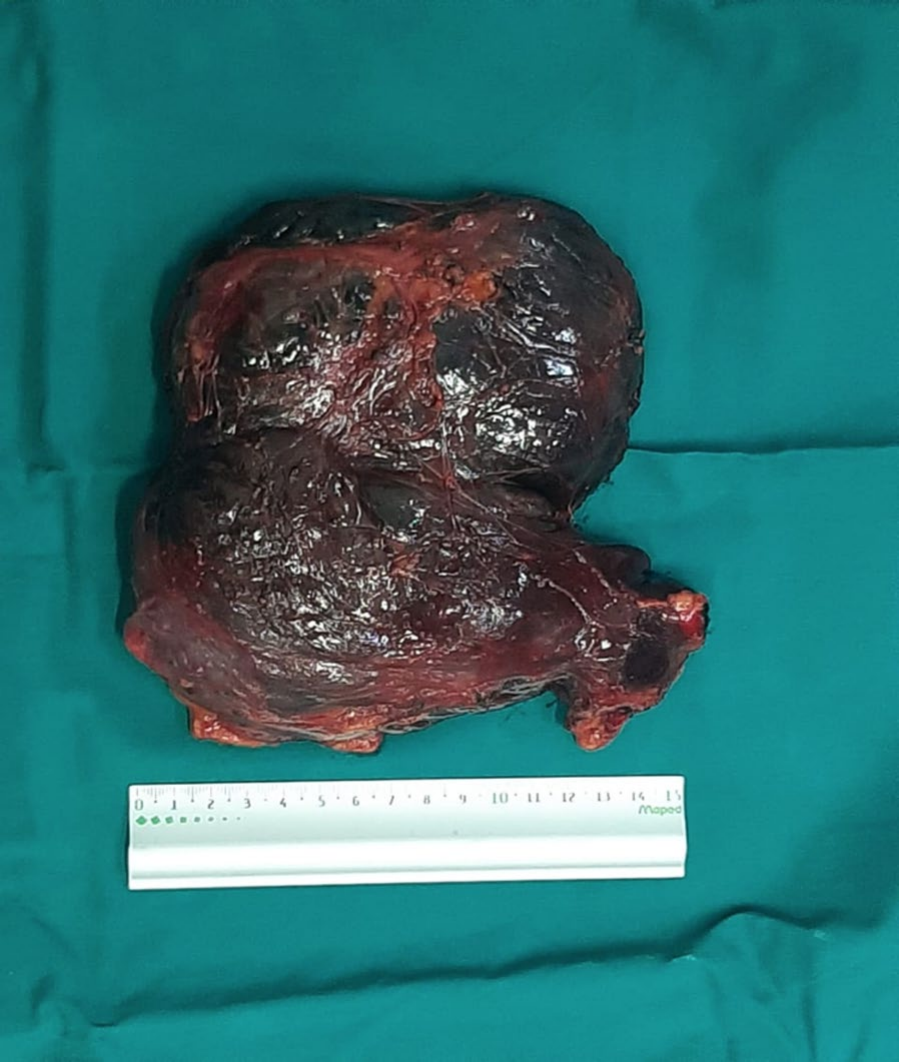
Photograph of resected specimen showing gray-red capsulated structure with focal areas of hemorrhage and necrosis

The tumor was completely resected *en bloc* without the removal of adjacent tissues. During the operation, her blood pressure and heart rate were quite stable. Following removal, it was identified that the tumor exhibited a capsule that was soft and gray-red (Fig 3). Hemorrhage, cystic degeneration, and necrosis were observed inside the tumors. On pathologic examination, tumor cells were oval or polygonal in shape and arranged in nests or trabeculae, containing rich cytoplasm with eosinophilic fine granules. Large nuclei were strongly stained and exhibited round or oval nucleoli. Tumor cells with deformed, large, or multiple nuclei were observed.

The postoperative course was uneventful, and the patient was discharged 7 days after the operation.

After 1 year of follow-up, the patient presented no relapse.

## Discussion

Since the COVID-19 pandemic has started, chest computer tomography (CT) has been used for diagnosis and follow-up in symptomatic patients suspected of COVID-19 infection [1]. Apart from its radiological features, the use of CT as a triage tool has unavoidably led to incidental findings [2], since not only lung parenchyma, but also surrounding extrapulmonary structures of the mediastinum, cardiovascular system, and upper abdomen are imaged. In our case, we reported an incidental finding of retroperitoneal paraganglioma in a patient with suspected COVID-19 pneumonia.

Paragangliomas are neuroendocrine tumors that arise from extra-adrenal chromaffin cells [3]. They can occur in four types of locations: branchiomeric, intravagal aorticosympathetic, visceral autonomic, and retroperitoneal paragangliomas. Retroperitoneal paragangliomas account for 1–3% of retroperitoneal tumors [4]. They affect mainly adults in the fourth or fifth decade of life, with no sex predilection [5].

Paragangliomas are classified into functional and nonfunctional [6]. Most patients with nonfunctional tumors are completely asymptomatic or may have pain and occasionally have symptoms of metastases. Functional tumors are easier to diagnose because urinary catecholamines are elevated. The typical presentation is hypertension. Other common symptoms include headache, diaphoresis, palpitations, and fever [7].

Initial evaluation involves 24-hour urine catecholamines and serum metanephrines. Radiological techniques, including CT and MRI, are useful for identifying and locating retroperitoneal paragangliomas. CT scan has a sensitivity of 95–100% and specificity of 67%. Some of the image characteristics of retroperitoneal paraganglioma were identified, including thick tortuous arteries and veins inside the tumor; strong enhancement in the arterial phase; tumor location close to the renal arteries and veins surrounding the abdominal aorta and inferior vena cava; and location of the tumor behind the inferior vena cava [3].

Cystic degeneration, necrosis, hemorrhage, and calcifications are common in retroperitoneal extra-adrenal paragangliomas but these imaging features tend to be observed in other retroperitoneal tumors [6].

Because of superior tissue characterization and absence of radiation hazards, MRI is recommended as the first-choice technique in evaluating patients with suspected paragangliomas. It also gives better information about the adjacent vascular structures [4].

Functional imaging techniques, including 123I-meta-iodobenzylguanidine (MIBG) scan and fluorine‑8‑l‑dihydroxyphenylalanine (18F-DOPA) positron emission tomography (PET) may be used to improve the sensitivity and specificity of diagnosis [3].

Surgery is the treatment of choice for paraganglioma [8], and it is associated with an improved survival rate, even in patients with distant metastasis. Complete surgical resection remains the only curative treatment but it is challenging, as these tumors are located near multiple vital blood vessels [9].

Before surgery, appropriate medical preparation with α-blocking and β-blocking agents is very crucial to avoid intraoperative hypertensive crisis [10].

Preoperative localized arterial embolization may help reduce blood loss during surgery. Adjuvant radiation therapy following surgery may improve median survival in malignant paraganglioma [11].

Long-term follow-up is very important following resection as patients can have persistent or recurrent disease or develop metachronous primary paraganglioma. In patients with metastatic disease, palliative chemotherapy with cyclophosphamide, dacarbazine, and vincristine is recommended.

## Conclusion

Paraganglioma is a rare pathological entity that occurs most often in young adults. The localization in the retroperitoneal region represents a therapeutic challenge. Preoperative diagnosis of paraganglioma is difficult owing to the lack of specific symptoms [12], but may be markedly improved by attaining the location and functional characteristics of the tumor, in combination with imaging results. Since COVID-19 started, the chest CT scan has become an essential tool used in disease staging. This has led to incidental findings of asymptomatic tumors and thus offered a curative treatment chance for patients.

## Data Availability

All the data was taken from the patient’s medical folder available at the archive of our institution.

## Abbreviations

CT: Computed tomography
PET: Positron emission tomography
